# Mammalian evolution and human mutation burden in Rab GTPases

**DOI:** 10.1016/j.bbrep.2026.102521

**Published:** 2026-02-27

**Authors:** Unmani Sidor, Graham M. Hughes, Jeremy C. Simpson

**Affiliations:** a School of Biology and Environmental Science, University College Dublin, Dublin 4, Ireland; b Cell Screening Laboratory, School of Biology and Environmental Science, University College Dublin, Dublin 4, Ireland

**Keywords:** Rab GTPases, Phylogenetics, Evolution, Cancer, Mutation

## Abstract

Rab GTPases are essential regulators of membrane trafficking with known roles in neurodegeneration, cancer progression and metastasis. While individually, Rabs have been extensively studied in disease and phylogenetic contexts, the overall evolutionary and mutation patterns that influence Rab function, at a family level, remain underexplored. Hence, we performed a family-wide investigation of Rabs in mammalian evolution integrated with disease-related mutations in modern humans. In this study, we analysed 54 Rab proteins across 62 placental mammals using branch-site models, to assess the extent of their sequence evolution across the mammalian lineages. We then combined these findings with the mutation data in human Rabs from UniProt. We defined three domain-level metrics, namely, constraint score, damage tolerance and overall mutation burden, to evaluate how Rab proteins as a whole, and in each of their domains, tolerate variation at the population level in humans. Our results suggest that the Rabs evolving across a larger number of mammalian species tended to show greater accumulation of damaging mutations in humans, particularly within the Switch I domain. This region is critical to Rab function and appears to exhibit cross-species variation while accommodating mutations relevant to human disease. The integrated framework used to examine mammalian Rab evolution and disease-related variation in human Rabs, can also be applied to investigate other conserved protein families implicated in diseases.

## Highlights

• We analysed 54 Rabs across 62 placental mammals assessing their sequence evolution.
• We defined 3 domain-level metrics evaluating how Rab proteins tolerate variation.
• Rabs 17, 33b, 34 and 36 show greater mammalian sequence evolution.
• Some human Rabs show greater accumulation of deleterious mutations.
• Our framework can be applied to examine other disease-implicated protein families.

## 1 Introduction

The ‘Ras-related in brain’ Rab GTPases are master regulators of intracellular membrane trafficking in eukaryotic cells [1,2]. Functioning as molecular switches, they alternate between a GTP-bound active state and a GDP-bound inactive state. With more than 60 members identified, Rabs play a central role in processes ranging from endocytosis, vesicle transport, to autophagy [3]. Disruption to Rab function, through dysregulation or coding mutations, has been implicated in a wide range of human diseases, including several cancers and neurodegenerative disorders [2,4]. For instance, aberrant expression of *RAB22A*, *RAB23*, and *RAB1A* has been associated with tumour progression [[5], [6], [7]], while mutations in *RAB32* and *RAB38* have been implicated in Parkinson's Disease [8,9]. Rab proteins are not only essential in the mature organism, but several studies have also highlighted their role in early embryonic development of model organisms such as mice (*M. musculus*), fruit flies (*D. melanogaster*) and nematodes (*C. elegans*) [10]. However, despite their well-established biological relevance, the collective evolutionary and mutation patterns that influence Rab function, at a family level, remain underexplored.

Generally, mutations or single-nucleotide polymorphism (SNPs), especially those located in conserved or functionally critical domains, can greatly affect the protein activity, often contributing to pathogenesis. For example, mutations in kinase domains, effector-binding domains and promoter elements have been implicated in impaired protein function and, consequently, disease onset [[11], [12], [13], [14]]. However, most studies examining mutations in Rab sequences have focused on individual proteins [8,15,16]. Considering the critical role of Rab dysfunction in disease incidence, there is a need to systematically investigate how the Rab family tolerates mutations across the various domains that are found in these proteins. Typically, Rab proteins consist of a GTP-binding region, Switch I/II domains and a hypervariable domain [17,18]. These regions govern nucleotide binding and effector interactions, and mutations within them can likely alter Rab function relevant to disease. Hence, examining mutations within these domains has the potential to reveal domain-specific variation that may contribute to disease progression.

The origin, evolution, and subfamily diversification of Rabs have been extensively examined, specifically ancestral complexity, species-specific Rab expansions/losses [19], and the contribution of Rabs to endocytic and exocytic processes in early eukaryotes [20]. More focused studies have examined the diversification of RAB7 and RAB9 subfamilies in vertebrates [21]. However, these studies do not explore Rab evolution in the context of disease susceptibility or mutation burden (level of damaging mutations or SNPs) relative to modern human populations. In this study, we address this gap by integrating evolutionary rate analyses of 54 Rabs across 62 placental mammals with domain-level mutation burden in their human orthologs. We combine comparative genomics analysis with human mutation data to identify key regions of adaptive flexibility and functional tolerance across Rab proteins.

One specific area of interest is the role of Rab evolution in mitigating cancer mortality risk, particularly in larger mammals which, despite their higher body mass and longevity, do not suffer from increased risks of cancer (e.g. as explained by Peto's paradox [22]). We therefore examined whether there is an evolutionary link between Rab evolution, body mass, and cancer mortality risk in mammalian species. Using published estimates of cancer mortality risk, body mass and lifespan [22], we performed an exploratory analysis of how Rab evolution relates to these species-level traits.

Our analyses suggest that *RAB36*, *RAB33B*, *RAB34*, and *RAB17* appear to be evolving across a large number of mammalian species. Other Rabs that also show a high evolutionary occurrence appeared to have more mutation burden in the Switch I domain of their human paralogs. Our findings highlight that the Switch I domain appears to exhibit cross-species variation while accommodating variation relevant to human disease. However, variation in this region did not show a significant association with the lifespan, body mass or cancer mortality risk of mammals. Overall, the study highlights the patterns of evolution within the Rab family. We present a combined approach to studying Rab evolution across mammals and their variation in humans, which can also be applied to investigate other conserved protein families implicated in diseases.

## 2 Results

### 2.1 The extent of Rab evolution varies across mammals

**Fig. 1:**
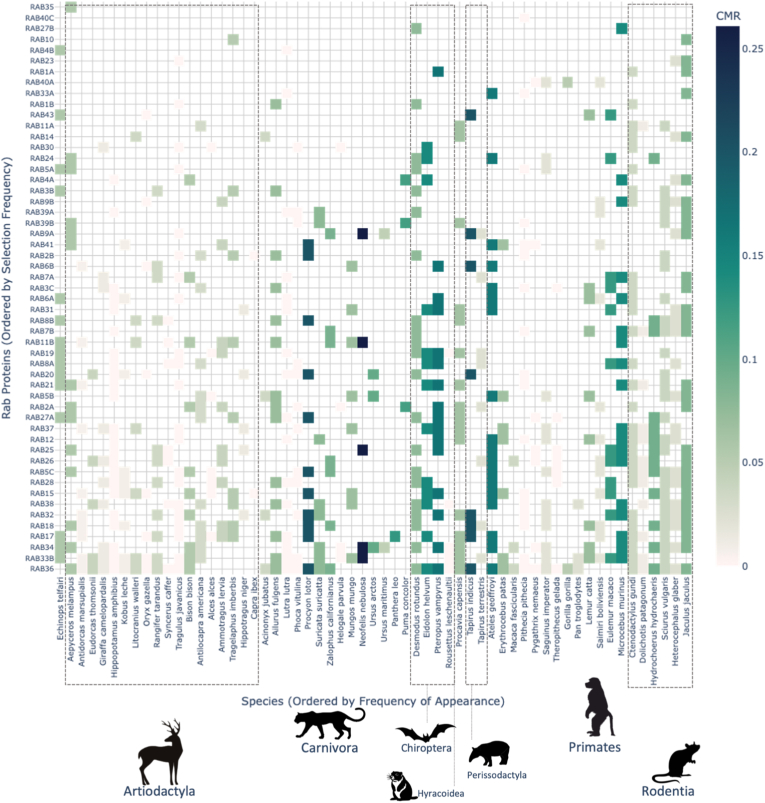
**Rab evolution across mammals.** The 53 Rabs showing positive selection in 62 placental mammals from 8 orders are displayed as a heatmap. The vertical axis indicates Rabs evolving across the lowest (top) to highest (bottom) number of species. Each vertical strip corresponds to a mammalian species, represented by a single colour reflecting the Cancer Mortality Risk (CMR) of the species, defined by Vincze et al. [22] Empty cells denote either a lack of significant selection or unavailable sequence data (see CMR distribution in Fig. S2).

A total of 54 Rabs were tested for positive selection across 62 mammals to assess the extent of their sequence evolution. Positive selection refers to the increase in frequency of favourable mutations (or alleles) in a gene that aids the fitness and survival of the species [22,23] (see Methods). Contrary to this is negative or purifying selection, where deleterious or harmful changes in gene sequences or amino acids are suppressed. While negative selection eliminates deleterious mutations to preserve essential function, positive selection highlights regions of adaptive change. This is particularly relevant to our study, which focuses on identifying such regions in the conserved Rab protein family that have undergone sequence-level changes. Of the 54 Rabs, 53 showed positive selection across placental mammals (Fig. 1). We defined the extent of positive selection by selection pressure score, i.e., the ratio of extant mammalian lineages showing positive selection. Species are grouped by mammalian order, depicting a varied range of Cancer Mortality Risk (CMR) scores among species [22]. As established by Vincze et al. [22], the CMR of a mammal is the ratio of cancer-related deaths and the total number of mammals assessed for their postmortem pathological records (see Methods). While our study does not calculate CMR directly, we incorporated species-level CMR values from Vincze et al. [22] to observe potential trends. Amongst the species included in our dataset, Artiodactyla generally exhibited lower CMR values than Carnivora, Primates and Chiroptera, consistent with the trends reported by Vincze et al. [22].

Of the 54 Rabs tested, 53 showed significant positive selection (FDR-corrected p < 0.05) in at least one mammalian lineage, with RAB22A the only one showing no selection across any species. Rab proteins with the highest rates of selection include RAB36, RAB33B, RAB34 and RAB17 (Fig. S1). Despite variability in cancer risk, lifespan, and body mass across mammals, these traits did not significantly correlate with the extent of Rab evolution (Spearman's correlation) (Figs. S3–S4). These results reveal lineage-specific patterns of Rab adaptation, with particular Rabs evolving independently across diverse mammalian taxa.

### 2.2 Mutation burden in human Rabs reflects mammalian evolutionary dynamics

**Fig. 2:**
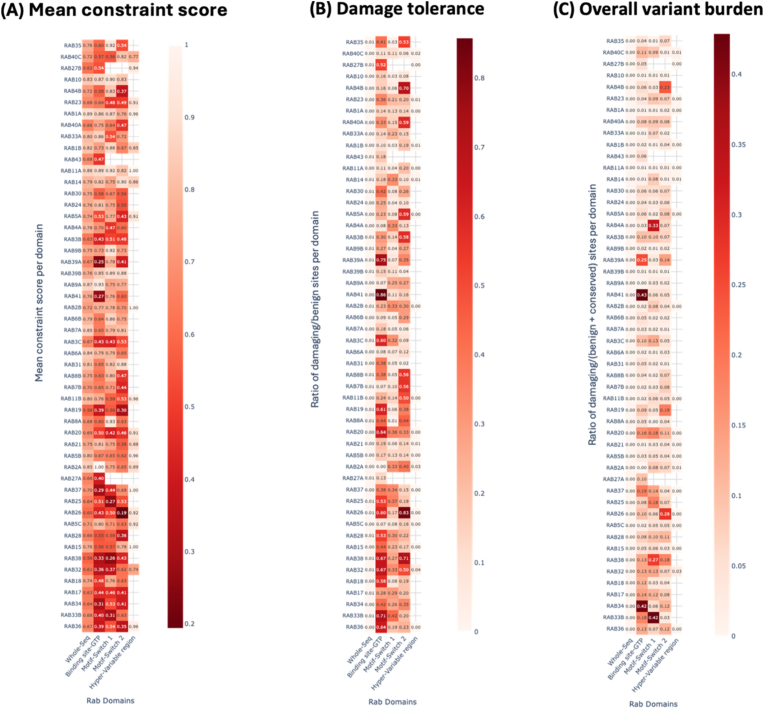
**Mutation burden in human Rabs across 3 metrics.** Heatmaps depicting the mutation burden in 49 human Rabs described using 3 metrics, (A) Mean constraint score, (B) Damage tolerance, and (C) Overall variant burden, are displayed. Each metric is length-normalised using the corresponding domain length and the Rabs are ordered (top to bottom) according to the number of mammals showing positive selection, as defined in Fig. 1. The X-axis represents the five protein domains examined (left to right): The entire Rab protein sequence, GTP-binding regions, Switch I and Switch II domains, and the hypervariable domain (see Table S1 for metric scores).

Next, we investigated whether Rab evolution across mammals corresponds to disease-relevant variation in humans. Specifically, we explored how the mammalian evolution of Rab sequences compared to the mutation burden in their human paralogs. Here, mutation burden refers to the relative accumulation of missense variants (SNPs) in the protein-coding sequences of human Rabs. These variants had either known disease associations or high damage prediction scores, such as Polyphen [23]. The Polyphen score provides an estimated probability of how damaging a missense mutation is to protein function. To quantify this mutation burden across 49 Rabs, three Rab-level metrics were defined: (1) constraint score, (2) damage tolerance, and (3) overall variant burden (Table 1) (see Methods). As detailed, the metrics were derived from curated UniProt annotations, incorporating both the known pathogenic mutations and the missense mutations predicted to be damaging based on PolyPhen [23] scores. Overall, these metrics assess the degree of mutation burden across the five most important Rab regions: the Switch I/II domains, the GTP-binding region, the hypervariable domain and the full-length Rab sequence. To account for differences in domain lengths, the metrics were normalised using two approaches, Length-Normalised (LN) scores and Proportion-Normalised (PN) scores. While both methods produced consistent patterns of metrics, LN scores offered more interpretable heatmaps with uniform scaling across domains (Fig. 2A–C). See PN-based scores in Fig. S5.

**Table 1:** **Summary of domain-level mutation burden metrics**. Each metric quantified the extent and nature of Across all three metrics, human Rabs corresponding to those under stronger positive selection in mammals exhibited greater localised mutation burden, particularly in key regulatory regions. This pattern was more pronounced in RAB4B and RAB26, with elevated damage tolerance and overall variant burden. For each metric, domain-specific scores were consistently higher than the full-length Rab scores, which was expected as computing scores across longer or full-length sequences can average and reduce the stronger signal localised in the functionally important regions. Amongst the domains, Switch I/II domains and the GTP-binding regions showed a higher concentration of missense mutations. The hypervariable domain showed relatively low scores across the metrics, which may reflect a reduced accumulation of mutations. However, this region is poorly annotated in databases, such as UniProt, likely due to its intrinsic sequence variability. Consequently, our interpretation of metric scores for this domain is restricted.

Metric	Definition	Normalisation method	Interpretation
**Mean constraint score** Min: 0.194 (RAB26) Max: 1 (multiple)	Average constraint score across all sites in the domain: 1 – (damaging/total variants at each site)	Inherently LN	Higher score implies stronger purifying selection (i.e., more conservation within the domain)
**Damage Tolerance** Min: 0 (multiple) Max: 0.857 (RAB41)	Ratio of damaging to benign sites in the domain	LN and PN	Higher score implies that the domain tolerates more damaging variation over benign
**Overall variant burden** Min: 0 (multiple) Max: (RAB41)	Ratio of damaging sites to benign and conserved sites	LN and PN	Higher score implies greater proportion of potentially deleterious variation in the domain

mutation burden across the domains of human Rabs. The maximum and minimum scores for each metric are also displayed, corresponding to the Rab(s) (see Table S1). The mean constraint score reflects the extent of conservation or purifying selection across all sites in a domain and is inherently normalised by length. Damage tolerance and overall variant burden compare the proportion of damaging to benign and/or conserved variants and were additionally normalised using two approaches: Length-Normalisation (LN) and Proportion-Normalisation (PN) (see Methods).

### 2.3 The switch I region acts as a focal point of mutation burden

The correlation between Rab evolution and the mutation burden was the strongest within two functionally critical regions – the Switch I domain and the GTP-binding motifs (Fig. 2A–C, Table S2). The degree of correlation slightly differed depending on the method used for normalising the mutation burden metrics. The PN scores retained a higher correlation across all metrics compared to the LN scores. However, for both LN and PN methods, Switch I metrics showed the strongest positive correlation with the number of mammalian species under positive selection (Table S2). By contrast, the GTP-binding region exhibited a weaker correlation (r ≈ 0.40, 0.0006 < p < 0.01) and significance in only two of the three metrics, irrespective of the normalisation method (Table S2). Hence, Switch I was prioritised for further domain-specific analyses. Although the Switch II domain also showed elevated metric scores in some Rabs, this signal was more sporadic and not consistently associated with Rab evolution across mammals. Since this study aimed to identify Rab domains that co-vary with mammalian evolution, Switch I remained the most informative domain for further investigation. Additional Rab domains showing a high concentration of predicted damaging variants include the GTP-binding sites of RAB41, RAB34, and RAB39A (Table S1). These domains are often involved in membrane targeting or effector specificity, and their elevated mutation burden may reflect lineage- or tissue-specific functional tuning [18].

### 2.4 Divergence in the switch I region reveals lineage-specific patterns

**Fig. 3:**
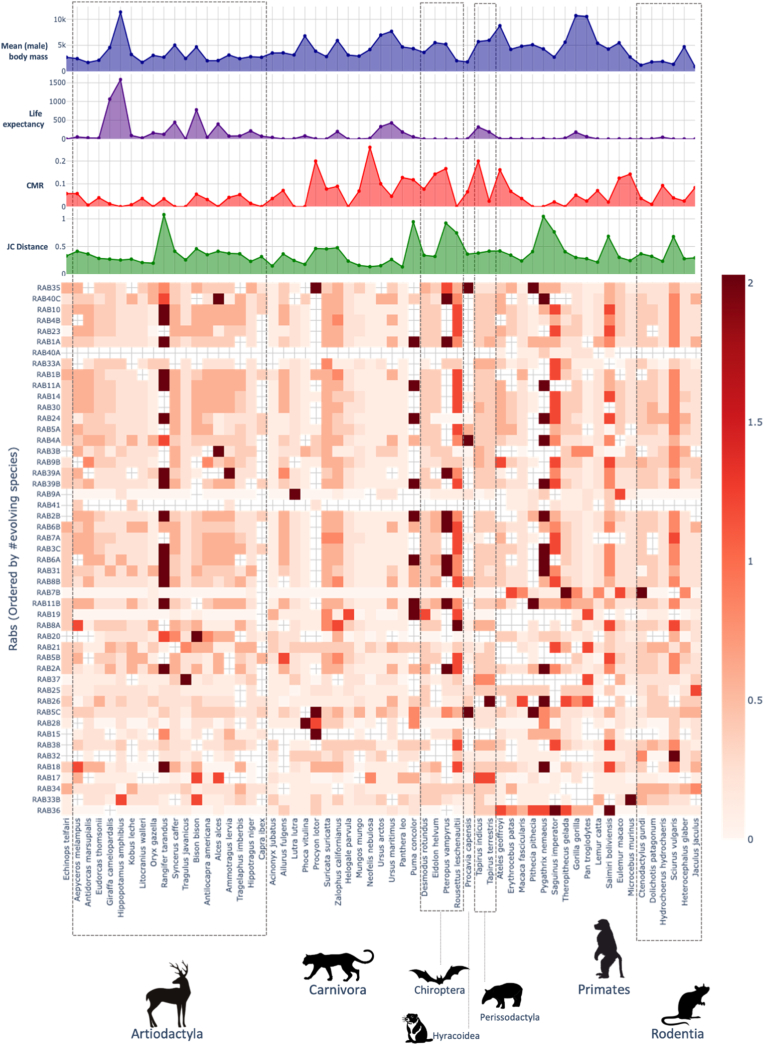
Divergence in mammalian Switch I region**s.** Heatmap showing the Jukes-Cantor (JC) distance between the Switch I region of each mammal relative to humans per Rab, indicated by colour. Rabs are ordered based on the rate of positive selection across species, i.e., from least (top) to most (bottom) evolving. Species are grouped per order. Mean male body mass, life expectancy (number of days after reaching sexual maturity), CMR, and JC distance values are also displayed.

While mutation burden captures how human Rabs accommodate variation across domains, it does not reflect how diverged these regions are across species. Aligning with the human-centric nature of the study, we aimed to understand how diverged the Switch I domain is between humans and other mammals. The rate of divergence in the Switch I regions across mammal species relative to humans was assessed using Jukes-Cantor (JC) distance, comparing each species to the human reference (Fig. 3). Here, the JC-distance represents the absolute divergence of the Switch I domains of mammalian species from humans, regardless of their rate and significance of evolution. Certain species, including *Pteropus vampyrus* (large flying fox), *Procyon lotor* (raccoon), and *Pygathrix nemaeus* (red-shanked douc), consistently exhibited high divergence from humans in the Switch I region across multiple Rabs (Table S3). These patterns suggest lineage-specific divergence in the Switch I domain, rather than isolated events of mutation.

To explore potential drivers of Switch I divergence, we tested whether JC-distance correlated with species-level traits, such as cancer mortality risk (CMR), body mass, and life expectancy. Across Rabs, JC-distance in Switch I did not show significant correlations with either CMR or body mass (Table S4). However, JC-distance was positively associated with species life expectancy for RAB36 (r = 0.4, p = 0.03) and RAB9A (r = 0.5, p = 0.001), while a negative correlation was observed for RAB34 (r = −0.49, p = 0.004).

Collectively, the results highlight the extent of Rab evolution across mammals, revealing both widely evolving and more conserved members of the family. From a human disease perspective, our findings demonstrate the domain-specific differences in how Rabs accumulate mutations within human populations.

## 3 Discussion

Our results show that *RAB36*, *RAB33B*, *RAB34* and *RAB17* have undergone sequence evolution in mammals through positive selection more frequently than any other genes within the Rab family. While case studies on the evolution of these specific Rabs are limited, it is worth noting their critical role in membrane trafficking. The paralogs RAB34 and RAB36, which localise at the Golgi apparatus, play an important role in ciliogenesis and in regulating the spatial distribution of late endosomes and lysosomes [[24], [25], [26]]. While loss-of-function mutations in *RAB34* are implicated in genetic disorders [27], RAB36 remains poorly characterised in the context of pathogenesis. Mutations in RAB33B, another Golgi-associated protein with a key role in autophagy, cause the skeletal disease Smith–McCort Dysplasia [16,28]. The epithelial-specific RAB17 is mostly localised at the plasma membrane and its up-regulation is linked to​ endometrial cancer and enhanced cell migration [[29], [30], [31]]. Hence, the adaptive pressures acting on these Rabs may be specific to their specialised roles in membrane trafficking.

Further, we assessed Rab evolution for association with broader species-level traits such as body mass and lifespan in the context of Peto's paradox. As demonstrated by Vincze et al. [22], Peto's paradox can be explained as follows: Larger mammals are not necessarily at greater cancer risk despite their higher body mass or longevity, owing to potentially higher accumulation of mutations. Although Peto's paradox serves as one possible context for our analysis, it is not necessarily a definitive test of cancer resistance in mammals. We did not find any association between Rab evolution and species-level traits, such as body mass and longevity, or between Rab evolution and species-level cancer mortality risk (CMR). Despite human Rabs being frequently implicated in cancer progression [1,4], most of the cancer-related dysregulations in human Rabs are due to altered expression, rather than coding mutations [2,32]. Therefore, the cancer risk in non-human mammals needs to be assessed additionally at the gene expression level. Together, these observations suggest that adaptation in mammalian Rabs does not seem to directly contribute to the cancer defence mechanisms or longevity of the species. Instead, it is possibly a result of other selective pressures yet to be determined.

When categorising missense variants as damaging or benign, we prioritised the use of Polyphen scores [23] when no disease information was associated. While these scores are predictive in nature and need to be interpreted with caution [33], they still offer a means of exploring mutational tolerance in human sequences. Within human Rabs, the Switch I, Switch II, and GTP-binding domains showed higher accumulation of mutations compared to the whole Rab sequence. Our damage tolerance metric revealed a stronger association between the Switch I domain and Rab evolutionary rate compared to damage tolerance measured across the full-length Rab sequence. This suggested a regional, rather than uniform tolerance. The Switch I region contributes to GTP-binding, mediating conformational change and interactions with Rab-binding proteins [18]. Hence, the tolerance in this region likely facilitates species-specific regulatory changes while maintaining the Rab GTPase function. It is important to note that what is classified as ‘damaging’ variants in humans may not be deleterious in other species. Currently, due to the limited availability of variant data across non-human mammals, we cannot yet determine whether the divergence of Switch I in non-human mammals reflects deleterious mutations or a species-specific adaptation as a response to its external selective pressures. As more cross-species variant datasets become available, future studies will be able to identify maladaptive mutations from lineage-species shifts. It is also important to note that the association between positive selection across mammals and mutation burden in humans should be interpreted cautiously. If a Rab domain can accumulate amino acid substitutions repeatedly across independent mammalian lineages without loss of function, this may indicate that the region tolerates the protein sequence change. Such tolerance could also permit the accumulation of missense variation within human populations. Our results are consistent with this possibility, although they do not establish a causal relationship between these evolutionary scales.

Together, these findings suggest that conserved regulatory protein families such as Rab GTPases may evolve through domain-specific flexibility. The Switch I domain in particular demonstrates this flexibility by having both a high mutational burden in humans and high rates of adaptive selection across different mammalian species. Our analyses did not detect a strong association between Rab evolution and species-level traits such as body mass, lifespan, or cancer mortality risk. This suggests that the sequence changes in Rabs are less likely to influence these traits directly. Instead, Rab evolution may reflect the adaptations in membrane trafficking pathways that occur alongside other genetic changes that are more directly linked to the broader species-level differences. Future studies examining Rab-interactors and related trafficking regulators may help in placing Rab evolution within the wider network of mechanisms influencing life- and cancer-related traits in mammals. Our integrative framework of evolutionary rate analysis and domain-level mutation burden may be extended to other Ras superfamily members or intracellular trafficking regulators to better understand how adaptive evolution occurs in conserved signalling networks.

## 4 STAR★Methods

Key resources table.

**Table:** 

REAGENT or RESOURCE	SOURCE	IDENTIFIER
Deposited data		
Data supported the key findings in this study.	This study	https://figshare.com/s/0aaabe1ef6b95c7ac243
**Software and tools**		
Tool to infer Orthologs from Genome Alignments (TOGA)	Kirilenko et al. [34]	https://github.com/hillerlab/TOGA
Python	Rossum & Drake [35]	https://www.python.org/
Aliview v1.3	Larsson [36]	https://github.com/AliView
AiStat v1.16	Wong et al. [37]	http://github.com/thomaskf/AliStat/
Clustal Omega	Sievers et al. [38]	http://www.clustal.org/omega/
PAL2NAL	Suyama et al. [39]	
HyPhy software suite	Kosakovsky Pond et al. [40]	https://hyphy.org/

## 5 Method details

### 5.1 Data mining and alignment

A total of 62 placental mammal species were chosen from 8 orders (Table S5) for comparative genomics analysis, reflecting a varying range of cancer mortality risk [22]. Specifically, the orders Artiodactyla (n = 18), Carnivora (n = 15), Primates (n = 15), Chiroptera (n = 6), Rodentia (n = 6), Perissodactyla (n = 2), Afrosoricida (n = 1), and Hyracoidea (n = 1) were represented in our dataset. While sampling depth differs within orders, this study does not aim to analyse order-level differences, but focuses on gene-level evolutionary patterns across all placental mammals. Sequence alignments of 54 Rab genes were generated using genomic information made available through the Zoonomia consortium (list of Rabs in Table S6). Briefly, for each available Rab gene, target mammal sequences were extracted from alignments that had previously been generated using the Tool to infer Orthologs from Genome Alignments (TOGA) [34], and subsequently realigned using Clustal Omega with translated amino acid sequences which were subsequently translated to codons using pal2nal [38,39]. These sequence alignments were used in all downstream analyses. As phylogenetic trees were needed for downstream selection analysis (see below), a Zoonomia mammal species tree was generated for each Rab gene analysed. This was done by taking the original tree of 471 mammals and clipping away taxa that were not included in a particular Rab alignment, using the *ape* package in R (v5.8.1) [41].

### 5.2 Selection tests

All Rab alignments were tested for the evidence of positive selection across mammals, where positive selection is inferred when a gene shows a higher rate of non-synonymous mutations per non-synonymous site (dN) than synonymous mutations per synonymous site (dS), generally calculated as dN/dS or ω > 1. Positive selection was inferred using both the adaptive Branch-Site Random Effects Likelihood (aBSREL) and Mixed Effects Model of Evolution (MEME) tests in the HyPhy software suite [40]. Specifically, mammalian lineages showing selection were identified through aBSREL and particular sites showing positive selection in the alignment were identified with MEME. The p-values from aBSREL test were corrected for multiple testing by taking all p-values for all species across all Rab genes and applying a False Discovery Rate (FDR) correction.

### 5.3 Measuring rates of selection pressure across Rabs

The FDR corrected p-values from the aBSREL were used to infer two metrics: *Selection Pressure* (SP) score and *Fisher's p-value*. The SP score was calculated as the ratio of evolving branches (p < 0.05) to the total number of tested branches for each Rab. Fisher's method for combining independent p-values was used to evaluate the overall evidence for selection across all branches of a Rab. This method calculates a test statistic as the sum of the negative log-transformed p-values, which is then compared to a chi-squared distribution with degrees of freedom equal to twice the number of branches. The resulting *Fisher's p-value* provides a single measure of whether the Rab is evolving collectively across the mammalian phylogeny. For the Rabs with multiple transcripts assessed in the aBSREL test, the transcript ID with the highest SP score and the lowest Fisher's P-values was selected for further analysis (all scores in Tables S7–S8, other species level information in Table S9). The number of top-evolving Rabs was decided based on the knee cut-off of the distribution of significantly evolving branches per Rab. The package kneed was used to identify the elbow of the distribution curve [42] (Fig. S1). For each Rab, Spearman and Pearson correlations were applied to test the association between the number of evolving branches and species traits, such as CMR, life span and body mass.

### 5.4 Human Rabs and domain-specific analysis/variant annotation and constraint scoring

Variant-level information of 49 human Rabs was obtained from UniProt, specifically the *variant* and *features* fields. For Rabs with available data, the amino acid sequences of the Switch I and Switch II domain, GTP-binding domain and hypervariable domains were identified and collated. For each site within the Rab sequences, the variant was labelled ‘damagingly variant’ if there was disease information in the ‘Disease association’ section of the variant entry in UniProt. For the variants with no disease association, Polyphen scores [23] listed in the UniProt variant viewer were used to measure the degree of damage from an amino acid change. While UniProt also provides SIFT (Sorting Tolerant From Intolerant) scores, Polyphen scores are more reliable when the protein of interest is well annotated [43]. Considering the relatively high coverage and annotation quality for Rab proteins on UniProt, Polyphen offered the most consistent integration with domain-mapped features. Additionally, even though Ensembl scores such as REVEL [44] and CADD [45] have been developed to increase predictive accuracy, they require harmonisation across databases and may not have been uniformly available for all Rab variants.

A site was labelled either ‘Conserved’ (no variants), ‘benignly variant’ (Polyphen score between 0 and 0.44) or ‘damagingly variant’ (Polyphen score >0.446). Each site was then assigned a constraint score (1 – damaging/total variants at the site). Further, 3 metrics were assigned to each full-length sequence of Rab and its domains:

(1) Overall constraint score: the mean constraint score across all sites
(2) Damage tolerance: the ratio of damaging/benign variants
(3) Overall mutation burden: the ratio of damaging variants/(benign variants + conserved sites)

The constraint score measures how tolerant a Rab sequence is to amino acid substitutions, with higher values indicating stronger purifying selection. Damage tolerance reflects the ratio of potentially damaging to benign variants, while overall mutation burden accounts for both benign variants and conserved sites, indicating the proportion of potentially deleterious changes. Together, these metrics provide a perspective on how each Rab and its domains tolerate variation at the population level. In cases of zero benign and/or conserved counts, the metrics damaging tolerance and overall mutation burden were adjusted by setting the denominator to 1, in order to avoid undetermined values, while still relaying information on the tolerance of the protein sequence (see Table S10 for all metric scores).

To account for differences in domain length, we applied two forms of normalisation:

(i) Length-Normalised scores, where the raw metric value was divided by the absolute length of the domain, and (ii) Proportion-normalised scores, where the raw metric is multiplied by the ratio of domain length and the total Rab sequence length.

The mean constraint scores were not normalised since this is inherently a length-normalised measure (Eq. (1)), as it normalises the domain as per its residue count. Hence, the above normalisation was applied to 2 metrics only: damage tolerance (Eq. (2)) and the overall mutation burden (Eq. (3)). Eqs. (1)–(3) depict the length-normalisation of the three metrics.

Eq. 1 $$Mean\;constraint\;score=\sum \left(1-\frac{Damaging\;variants}{Total\;variants\;at\;the\;site}\right)/Domain\;length$$

Eq. 2 $$\text{Damage}\;\text{tolerance}=\left(\frac{Damaging\;sites\;per\;domain}{Benign\;sites\;per\;domain}\right)\;/\text{Domain}\;\text{length}$$

Eq. 3 $$\text{Overall}\;\text{mutation}\;\text{burden}=\left(\frac{Damaging\;sites\;per\;domain}{Benign\;and\;conserved\;sites\;per\;domain}\right)\;/\text{Domain}\;\text{length}$$

### 5.5 Divergence of switch I domain in humans (Jukes-Cantor distance)

Aliview v1.3 was used to view the codon alignments of Rab sequences and extract the Switch I regions identified via UniProt (Switch I sequence information in Table S11). For each Rab, the pairwise proportion of nucleotides differing between species (uncorrected p-distance) for the switch I region was obtained using AliStat v1.16. All p-distances between each mammal and the human ortholog for each Rab gene were extracted and used to explore evolutionary divergence in the switch I domain. Using these p-distances, the Jukes-Cantor (JC) evolutionary distance for each Switch I region between all combinations of species was then calculated as per Eq. (4) [46,47].

Eq. 4 $$D_{\text{JC}}=-3/4\;*\;\ln\left(1-4/3\;*\;p\right)$$

The JC distances were not reported for sequence pairs with p-distance >0.75 due to undefined JC distance values, i.e., when (1 - p*4/3) < 0 (see Table S9). Plotly was subsequently used for data visualisation.

## Lead contact

Further information and requests for additional data should be directed to and will be fulfilled by the lead contact, Prof. Jeremy Simpson (jeremy.simpson@ucd.ie).

## Materials availability

This study did not generate new, unique reagents.

## Author contributions

UJ, GMH, and JCS conceived the project and developed the initial project workflow. UJ and GMH acquired and cleaned the data, followed by data analyses. GMH and JCS contributed to project administration and supervision. All authors contributed to the first draft and the revisions.

## Declaration of competing interest

The authors declare no competing interests.

## Data Availability

Data used in the statistical analysis of this study have been deposited in Figshare (https://figshare.com/s/0aaabe1ef6b95c7ac243).
