# Supplementary material for: Mammalian evolution and human mutation burden in Rab GTPases
Source: Biochem Biophys Rep. 2026 Feb 27;45:102521. doi: 10.1016/j.bbrep.2026.102521 (PMC12966740; doi:10.1016/j.bbrep.2026.102521)
Supplement: Multimedia component 2 [file mmc2.docx]

**Supplemental Figures**

**Mammalian evolution and human mutation burden in Rab GTPases**

**Unmani Sidor^1,2^, Graham M. Hughes^1,**^, Jeremy C. Simpson^1,2,**,***^**

**^1^School of Biology and Environmental Science, University College Dublin, Dublin 4, Ireland**

**^2^Cell Screening Laboratory, School of Biology and Environmental Science, University College Dublin, Dublin 4, Ireland**

**^**^Second listed corresponding author**

**^***^Corresponding author;** [**jeremy.simpson@ucd.ie**](mailto:jeremy.simpson@ucd.ie)

**1. Top evolving Rabs**


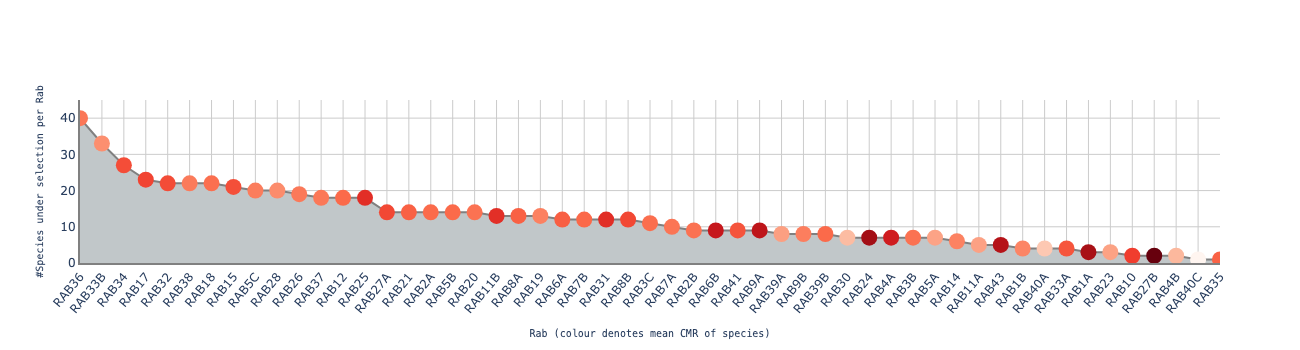


***Figure S1.*** *The distribution curve shows the count of mammals under positive selection for each Rab, coloured by the mean Cancer Mortality Risk (CMR) of the evolving species.*

Python package *kneed^1^* was used to identify the top N Rabs undergoing positive selection. The sensitivity was tested at various values of N (see below). With maximum point of curvature being at x=4, the top 4 Rabs above this knee-point were referred to as the most evolving across the studied mammals. These include RAB36, RAB33B, RAB34, and RAB17.

| **Sensitivity** | **Elbow of the curve (x)** |
| --- | --- |
| 0.5 | 4 |
| 1 | 4 |
| 1.5 | 4 |
| 2 | 14 |
| 5 | None/ undefined |
| 10 | None/ undefined |

**2. Distribution of Cancer Mortality Risk (CMR)**


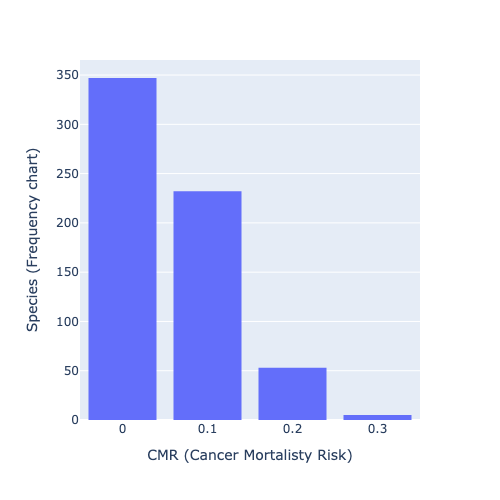


***Figure S2.*** *The distribution of CMR values across 62 species selected from Vincze et al.^2^*

**3. Correlating CMR to the extent of Rab evolution**


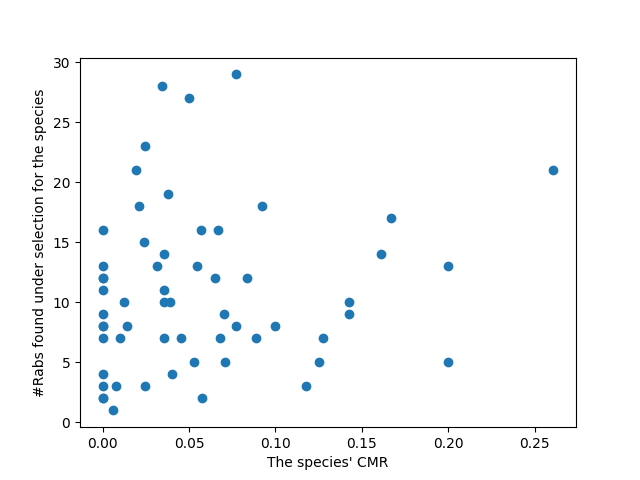


***Figure S3.*** *The X-axis displays the CMR of a total of 62 species, while the Y-axis represents the number of Rabs under positive selection within the species. The correlation coefficients across the two axes are 0.147 (Spearman) and 0.139 (Pearson).*


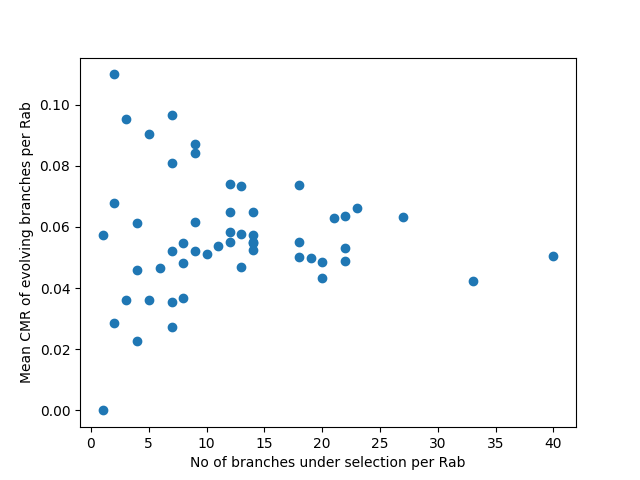


***Figure S4.*** *The X-axis displays the count of species under positive selection for each Rab. The Y-axis displays the mean CMR of the evolving species for the respective Rab. The correlation coefficients associating the two values are 0.068 (Spearman) and -0.014 (Pearson).*

**4. Proportion Normalised Metric scores**


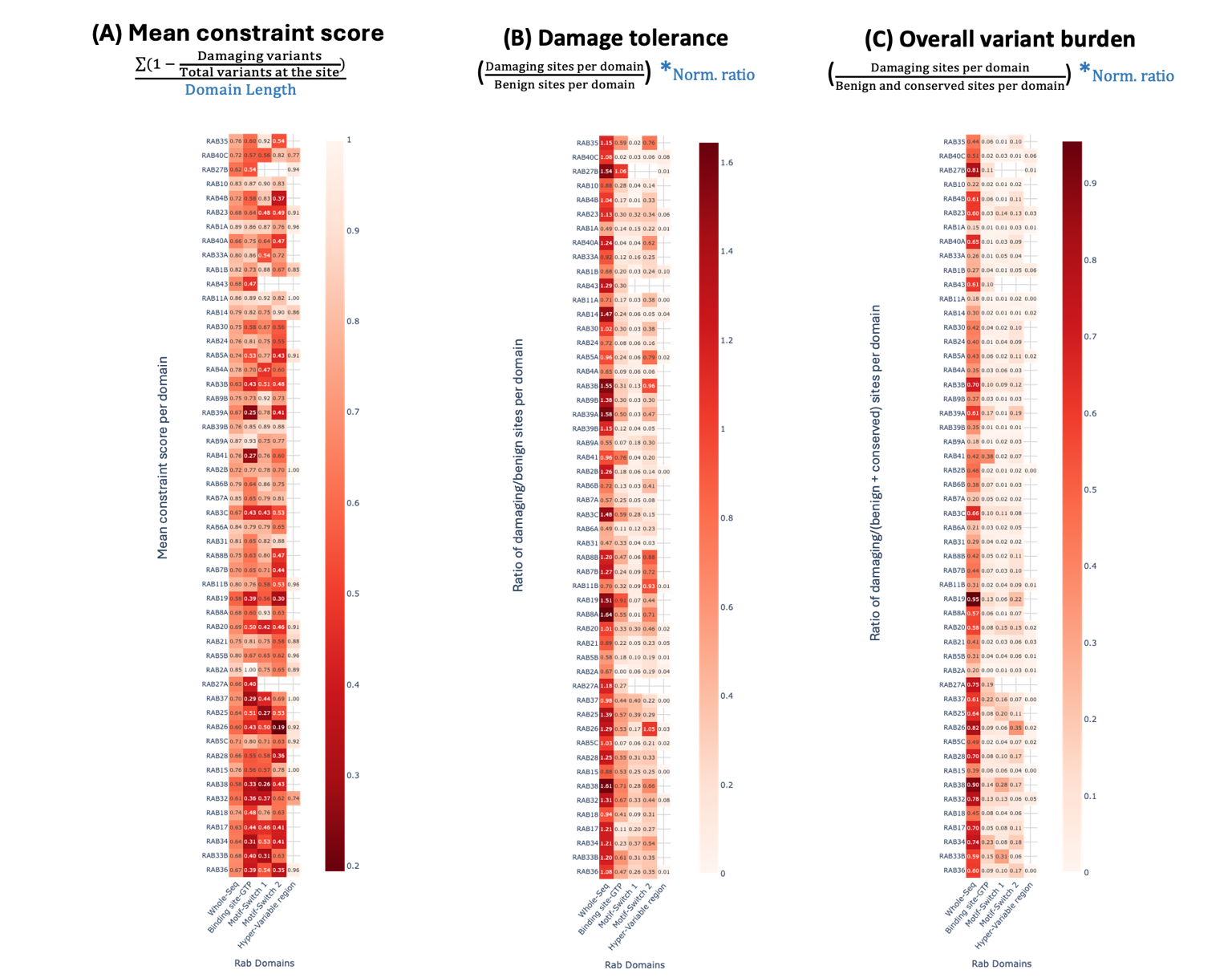


***Figure S5.*** ***Mutation burden in human Rabs across 3 metrics.*** *Heatmaps depicting the mutation burden in 49 human Rabs described using 3 metrics, (A) Mean constraint score, (B) Damage tolerance, and (C) Overall variant burden, are displayed. Each metric is proportion-normalised using the corresponding normalisation ratio, as indicated by the denominator (blue) in the formula displayed above each heatmap. This ratio is the proportion of domain length to the full-sequence length of the corresponding Rab. Rabs are ordered (top to bottom) according to the number of mammals showing positive selection, as defined in Fig. 1. The X-axis represents the five protein domains examined (left to right): The entire Rab protein sequence, GTP-binding regions, Switch I and Switch II domains, and the hypervariable domain (see Table S1 for metric scores).*

**References**

1. Satopaa V, Albrecht J, Irwin D, Raghavan B. Finding a ‘Kneedle’ in a Haystack: Detecting Knee Points in System Behavior. In: 2011 31st International Conference on Distributed Computing Systems Workshops [Internet]. Minneapolis, MN, USA: IEEE; 2011 [cited 2025 Dec 8]. p. 166–71. Available from: <http://ieeexplore.ieee.org/document/5961514/>

2. Vincze O, Colchero F, Lemaître JF, Conde DA, Pavard S, Bieuville M, et al. Cancer risk across mammals. Nature. 2022 Jan 13;601(7892):263–7.
